# Impact of an Aluminization Process on the Microstructure and Texture of Samples of Haynes 282 Nickel Alloy Produced Using the Direct Metal Laser Sintering (DMLS) Technique

**DOI:** 10.3390/ma16145108

**Published:** 2023-07-20

**Authors:** Jarosław Mizera, Bogusława Adamczyk-Cieślak, Piotr Maj, Paweł Wiśniewski, Marcin Drajewicz, Ryszard Sitek

**Affiliations:** 1 Faculty of Materials Science and Engineering, Warsaw University of Technology, Wołoska 141, 02-507 Warsaw, Poland; 2 Department of Materials Science, Faculty of Mechanical Engineering and Aeronautics, Rzeszów University of Technology, Al. Powstańców Warszawy 12, 35-959 Rzeszów, Poland

**Keywords:** Haynes 282 nickel alloy, DMLS, texture, CVD, aluminide layer

## Abstract

In this study, we examined the effects of an aluminization process on the microstructure and texture of Haynes 282 nickel samples fabricated using the direct metal laser sintering technique. The aluminization process involved the use of chemical vapor deposition with AlCl_3_ vapors in a hydrogen atmosphere at a temperature of 1040 °C for 8 h. Following the 3D printing and aluminization steps, we analyzed the microstructure of the Haynes 282 nickel alloy samples using light microscopy and scanning electron microscopy. Additionally, we investigated the texture using X-ray diffractometry. A texture analysis revealed that after the process of direct laser sintering of metals, the texture of the Haynes 282 nickel alloy samples developed a texture typical of cast materials. Then, in the aluminization process, the texture was transformed—from foundry-type components to a texture characteristic of recrystallization.

## 1. Introduction

Haynes 282 is a nickel alloy that is widely used in the aerospace industry because of its outstanding mechanical properties at high temperatures [1], which are mainly attributed to the strengthening effect of the γ’ phase and solution hardening. Both of these mechanisms significantly decrease the mobility of dislocations at high temperatures [2]. The alloy has a large content of Cr and other refractory metals, which increases its oxidation resistance and chemical stability [3]. Thanks to these advantages and its relatively low price, Haynes 282 is commonly used in demanding applications. Laser powder bed fusion (LPBF) is a modern process that involves sintering metallic powders, layer by layer, using a high-power laser [4]. The process is conducted in a protective atmosphere, which decreases oxidation and contamination of the powder. The main advantages of this method are tool-free machining to the desired shape and size, relatively high strength, and the ability to machine materials that can be problematic using conventional manufacturing methods. The disadvantages are the relatively high costs of the equipment and the powder material, long manufacturing times, and a non-equilibrium structure that is susceptible to cracking and porosity defects [5]. There is a plethora of research concerning an enormous variety of materials produced this way; they include nickel alloys, steels, titanium, composite refractory materials, and others [6,7,8]. The laser powder bed fusion (LPBF) of nickel-based alloys is a widely used process that results in a characteristic microstructure with dendritic meltpools. Phase segregation is an undesirable feature that decreases a material’s wear properties at high temperatures, which is especially unacceptable for uses in aviation [9]. As a remedy, heat treatment is used. The material is supersaturated and aged at a specific temperature for a defined length of time, depending on the alloy. It is worth noting that the primary structure after LPBF significantly affects the properties after heat treatment as well [10]. In numerous research papers, the key mechanical properties are significantly lower than the wrought material. This is most probably a result of defects and heterogeneities imposed in the LPBF [11,12,13]. For this reason, additional treatments are used to increase the resistance of the material [14,15,16].

One promising avenue for advancing the structural integrity and surface properties of nickel alloys fabricated through 3D printing techniques is the implementation of high-temperature thermo-chemical treatments. These treatments aim to enhance structural homogeneity while improving surface characteristics. By employing surface engineering techniques to create corrosion-resistant layers at elevated temperatures, the range of applications for nickel alloys manufactured using additive techniques can be significantly expanded, particularly in the demanding high-temperature environments seen in the energy and aviation industries. In this context, the β-NiAl intermetallic phase presents a particularly advantageous set of properties, including a high melting point, low density, and high elasticity modulus [17,18]. Exploiting these properties, the objective of this study was to investigate the effects of a high-temperature, low-activity aluminization process on the microstructure and texture of Haynes 282 nickel alloy samples produced using the direct metal laser sintering (DMLS) technique.

The aim of this study was to investigate microstructural changes resulting from the aluminization process. It is widely recognized that surface structure plays a significant role in chemical vapor deposition (CVD). In this research, our main focus was on analyzing the texture of the resulting layer and its impact on the morphology of both the layer itself and the 3D-printed material obtained through direct metal laser sintering (DMLS). Remarkably, there is a lack of previous research that specifically examines the use of a combination of aerospace materials in such a context.

By examining the microstructural changes induced by the aluminization process, we sought to gain insight into the effects of surface structure on CVD. We investigated both the surface texture of the layer and the characteristics of the DMLS printed material. Our study seeks to bridge the gap in the existing literature by providing a comprehensive analysis of the combined application of aerospace materials in this particular context.

## 2. Materials and Methods

### Sample Preparation and Thermo-Chemical Treatment

**Figure 1:**
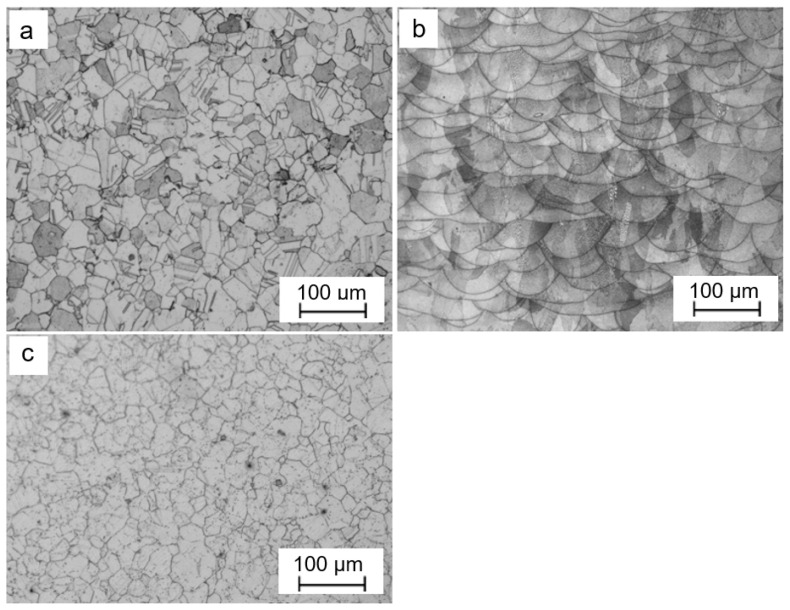
Microstructure of Haynes 282 samples: as received state—(**a**); produced using DMLS in the Y-Z plane—(**b**); annealed at 1040 °C for 8 h—(**c**).

Samples 20 × 20 × 20 mm in size were produced from a powder of Haynes 282 nickel alloy, obtained from the company Höganäs for the EOS M 100 printer operating in DMLS technology. The process of producing the sample was carried out using the following parameters: laser power P—80 W; laser velocity V—800 mm/s; distance between successive paths H—0.05 mm; layer thickness D—0.02 mm. After the 3D printing process, the surfaces of the samples were polished with grade 800 sandpaper. Then, 12 h before the aluminization process, the surfaces of the samples were sandblasted to activate them (using particles of α-Al_2_O_3_ powder) and then cleaned in an ultrasonic washer in ethyl alcohol. On those as-prepared surfaces, we carried out a low-activity process of aluminization using the chemical vapor deposition (CVD) in AlCl_3_ vapors in an atmosphere of hydrogen as the carrier gas, at a temperature of 1040 °C for 8 h. Furthermore, it is important to highlight that the temperature falls within a range suitable for solution heat treatment. This specific heat treatment process is designed to promote a homogenization of the materials. The metallographic specimens to be investigated were polished with sandpaper of grades 320, 600, and 1200 and then further polished using diamond suspensions of grade 3 μm, followed by 1 μm. The samples for the microstructural observations were etched in Kalings chemical reagent. The observations were made using a Zeiss Axio Scope A1 metallographic microscope and a Hitachi TM-1000 scanning electron microscope. A quantitative analysis of the texture was performed on the basis of four incomplete pole figures (1 1 1), (2 0 0), (2 2 0), and (3 1 1). The measurements were made using a Bruker D8 Discover X-ray diffractometer using filtered CoKα radiation and a spot beam about 1 mm in diameter. Based on the pole figures measures, orientation distribution functions (ODF) were calculated for each sample, and the contributions of the main texture components were quantified.

## 3. Research Results and Discussion

### 3.1. Microstructure

Figure 1 shows the microstructure of the Haynes 282 nickel alloy samples in the initial state, as well as after being produced using the EOS M 100 printer operating in DMLS technology. In the initial state (Figure 1a), within the microstructure of the Haynes 282 alloy, there are visible grains of austenite and twins. The microstructure of the sample produced in the Y-Z building plane (Figure 1b) consists of characteristic layers of melt pools. The visible melting pools overlap one another, leaving no empty spaces between them, which suggests that the samples produced have strong mechanical properties. The resulting microstructure is typical of nickel alloys produced using 3D printing.

For comparison, a 3D-printed Haynes 282 sample was subjected to a heat treatment process resembling aluminization, at a temperature of 1040 °C for 8 h. Surprisingly, the results exhibit striking similarities, indicating that temperature and diffusion play pivotal roles in shaping the final microstructure. Notably, both cases demonstrated a distinct influence of recrystallization, thereby highlighting its significance in the observed phenomena. The microstructures obtained from these experiments are visually depicted in Figure 1a,c for ease of reference and analysis.

**Figure 2:**
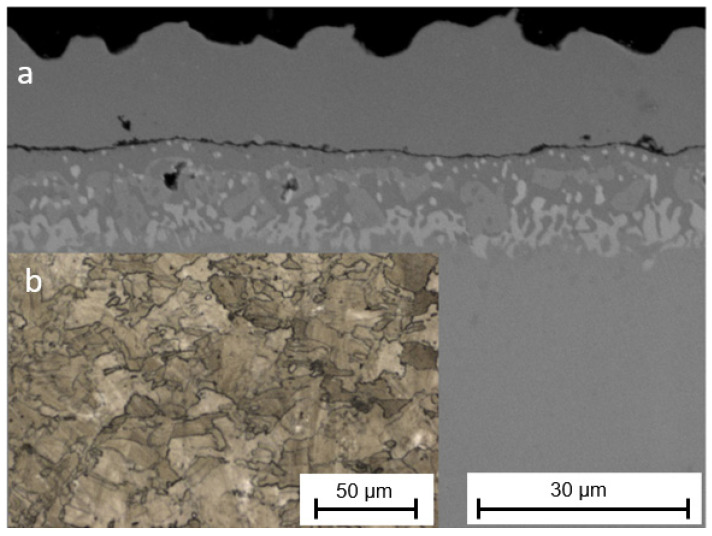
Cross-section of Haynes 282 nickel alloy produced using DMLS and subjected to an aluminization process: microstructure of the layer—(**a**); microstructure of the substrate—(**b**).

A cross-section of the layer created during the CVD process is shown in Figure 2a. The layer has a complex structure in which two main zones can be distinguished: an exterior β-NiAl zone about 14 µm thick and an interior zone about 15 µm thick, formed of precipitates rich in chromium and other elements of the substrate. The sublayers are divided by a thin zone of Al_2_O_3_ (residue from the sandblasting process). The arrangement of the layers is typical for the process of aluminizing nickel alloys using the CVD method with AlCl_3_ vapors [19,20]. The microstructure of the substrate after the aluminization process is presented in Figure 2b. No melting pools characteristic of the DMLS process are visible; the material has undergone recrystallization, and grain boundaries have appeared.

### 3.2. Texture

#### 3.2.1. Texture of Haynes 282 Nickel Alloy Samples in the Initial State (as Received from the Producer)

**Figure 3:**
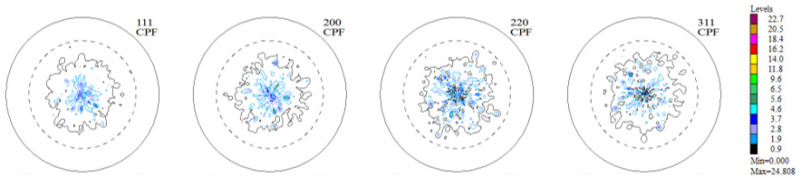
Experimental, incomplete pole figures (1 1 1), (2 0 0), (2 2 0), and (3 1 1) for the sample of Haynes 282 in the initial state.

**Figure 4:**
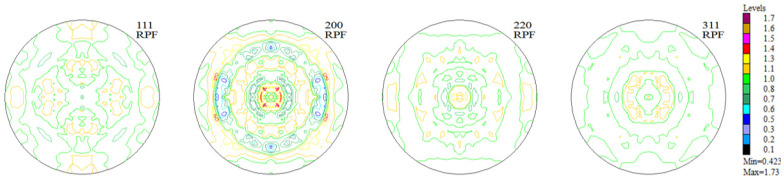
Complete pole figures (1 1 1), (2 0 0), (2 2 0), and (3 1 1) for the sample of Haynes 282 in the initial state.

**Figure 5:**
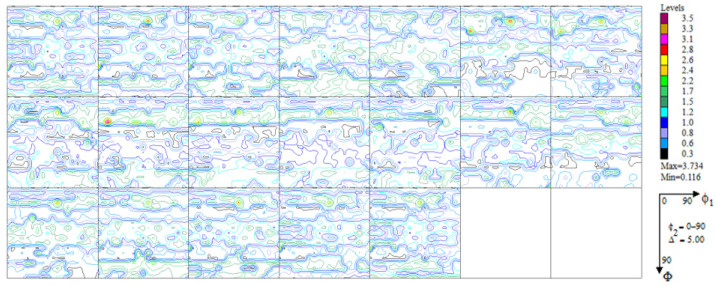
Orientation distribution functions maps for the sample of Haynes 282 in the initial state.

The experimental, incomplete pole figures (IPF), complete pole figures (CPF), and orientation distribution functions (ODF), along with the identified contributions of particular texture components for the Haynes 282 nickel alloy sample in the initial state, are presented in Figure 3, Figure 4 and Figure 5 and Table 1. The image of the initial texture revealed in the CPF and ODF, presented in Figure 5, clearly indicates the axial character of the grain orientations identified in the sample after casting and thermal treatment—they are dispersed around the <1 0 0> fiber, which is characteristic of casting states.

#### 3.2.2. Texture of Samples of Haynes 282 Nickel Alloy Produced Using the DMLS Technique (in the Y-Z Sample Building Plane)

**Figure 6:**
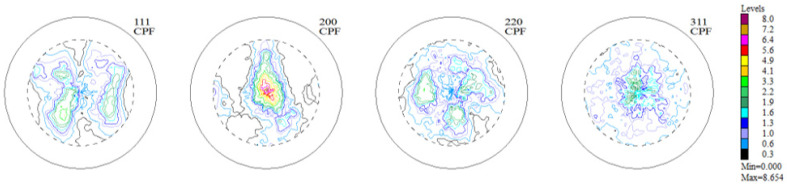
Experimental, incomplete pole figures (1 1 1), (2 0 0), (2 2 0), and (3 1 1) for the sample of Haynes 282 produced using DMLS.

**Figure 7:**
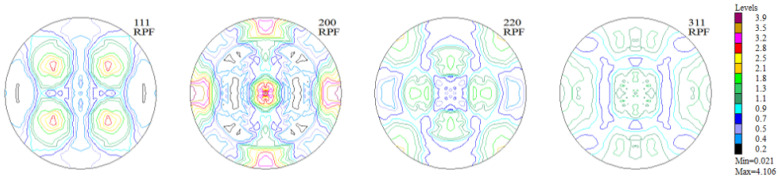
Complete pole figures (1 1 1), (2 0 0), (2 2 0), and (3 1 1) for the sample of Haynes 282 produced using DMLS.

**Figure 8:**
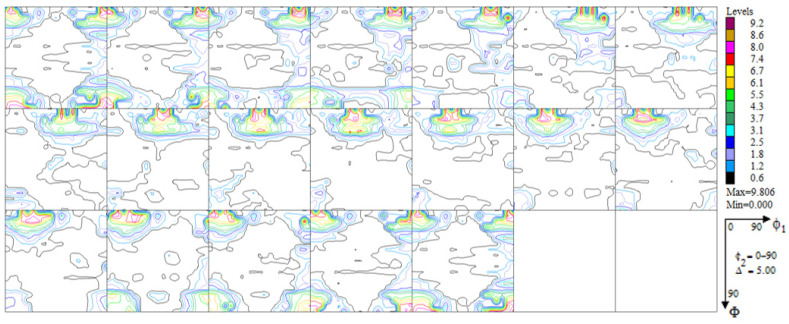
Orientation distribution function maps for the sample of Haynes 282 produced using DMLS.

The measurements of texture after the printing process were carried out in the Y-Z sample building plane. The incremental printing of the alloy under study resulted in a sharp (clear) texture in 2/3 of the volume, with two components (Figure 6 and Figure 7, Table 2). In more than half of the volume of the samples investigated, a cubic texture formed (0 0 1) <1 0 0>, as is characteristic of printed materials. In turn, in about 10% of the volume of the material a texture component was identified that is also present in the non-printed material (Figure 8)—in the as-delivered (reference) state, that is (4 1 1) <2 5 $\bar{3}$>.

#### 3.2.3. Texture of Samples of Haynes 282 Nickel Alloy Produced Using the DMLS Technique (in the Y-Z Sample Building Plane) and after the Process of Aluminization

**Figure 9:**
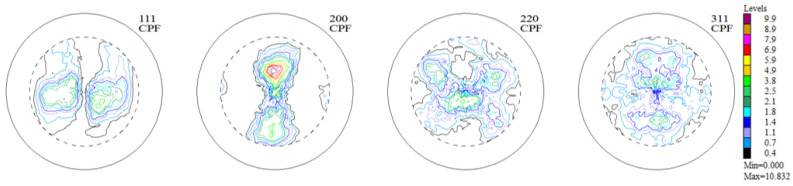
Experimental, incomplete pole figures (1 1 1), (2 0 0), (2 2 0), and (3 1 1) for the substrate of the sample of Haynes 282 produced using DMLS and after the process of aluminization (CVD).

**Figure 10:**
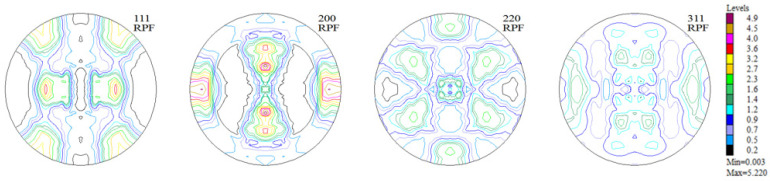
Complete pole figures (1 1 1), (2 0 0), (2 2 0), and (3 1 1) for the substrate of the sample of Haynes 282 produced using DMLS and after the process of aluminization (CVD).

**Figure 11:**
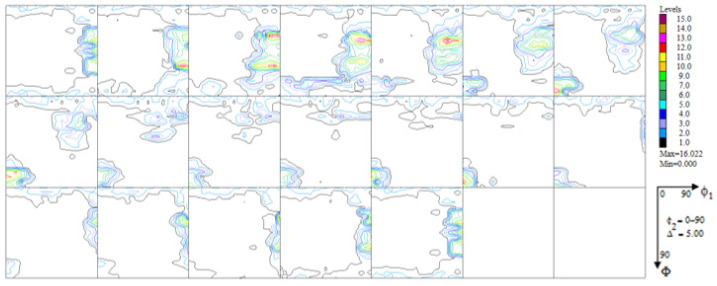
Orientation distribution functions for the substrate of the sample of Haynes 282 produced using DMLS and after the process of aluminization (CVD).

A significant reconstruction of the texture took place as a result of the thermo-chemical treatment applied to the printed material (the tests were conducted on the substrate without the layer). The texturing extended throughout almost the whole volume of the material (only about 7% of the volume was occupied by grains having a random orientation—Figure 9). Among the six texture components identified, two took up half the volume: ~(4 3 1) <3 $\bar{4}$ 0> and ~(1 5 3) <0 $\bar{3}$ 5>, around (0 1 1) <0 1 1> (~12% vol.). In short, one may acknowledge that almost 2/3 of the volume of the material accepted an orientation focused around (h k l) <1 1 0>. An exact analysis of the texture components as they emerged after the aluminization process (Figure 10, Table 3) showed that a large fraction of the volume of the material (25%) was occupied by grains dispersed around the orientation (h k l) <2 1 0>—the orientations (0 0 1) <2 $\bar{1}$ 0> and (1 0 2) < $\bar{2}$ 0 1>. This means that in almost all of the volume of the material produced using DMLS and subjected to the process of aluminization, grains dominated, whose orientations were dispersed around the components (h k l) <1 1 0> and (h k l) <2 1 0>, which is characteristic of textures after recrystallization (see Figure 11).

## 4. Conclusions

The utilization of DMLS (direct metal laser sintering) with Haynes 282 nickel alloy powder enables the production of samples with exceptional qualities, including a fine-crystalline structure and minimal porosity. This demonstrates the viability of DMLS as a reliable technique for fabricating high-quality metal components.

After the additive manufacturing process of the Haynes 282 alloy, a texture similar to that of the cast materials developed. In the printed material, however, a fine-crystalline structure was obtained. This underlines the importance of 3D printing in terms of optimizing the microstructure of materials, which leads to an improvement in their performance properties.

The process of aluminizing the Haynes 282 alloy produced by the DMLS technique leads to the transformation of the casting-type texture (initial state before the CVD process) to the recrystallization characteristic after the layer deposition process. This transformation is primarily attributed to the elevated temperatures associated with the CVD process, which leads to recrystallization in the substrate.

## Figures and Tables

**Table 1:** Volume fraction of the main texture components in the sample of Haynes 282 in the initial state.

Texture Component {h k l} <u v w>	Volume Fraction [%]
(4 1 1) <2 5 $\bar{3}$>	5.0
Background	95.0

**Table 2:** Volume fraction of the main texture components in the sample of Haynes 282 produced using DMLS.

Texture Component {h k l} <u v w>	Volume Fraction [%]
(0 0 1) <1 0 0>	54.0
(4 1 1) <2$\;5\;\bar{3}$>	10.0
Background	36.0

**Table 3:** Volume fraction of the main texture components in the sample of Haynes 282 nickel alloy produced using DMLS and after the process of aluminization (CVD).

Texture Component {h k l} <u v w>	Volume Fraction [%]
(4 3 1) <3$\;\bar{4}$ 0>	27.0
(1 5 3) <0$\;\bar{3}$ 5>	23.0
(0 0 1) <2$\;\bar{1}$ 0>	14.0
(0 1 1) <0 1 1>	12.0
$(1\;0\;2)\;<\bar{2}$ 0 1>	11.0
$(4\;\bar{1}$$1)\;<2\;5\;\bar{3}$>	6.0
Background	7.0

## Data Availability

Data are available from the first author and can be shared with anyone upon reasonable request.
